# The Effects of City-County Mergers on Urban Energy Intensity: Empirical Evidence from Chinese Cities

**DOI:** 10.3390/ijerph18168839

**Published:** 2021-08-22

**Authors:** Zhi Li, Ruyi Yan, Zuo Zhang, Yue Sun, Xiaogang Zhang

**Affiliations:** 1 School of Public Administration, Xi’an University of Architecture and Technology, Xi’an 710055, China; yanruyi@xauat.edu.cn (R.Y.); sunyue@xauat.edu.cn (Y.S.); zhangxg@xauat.edu.cn (X.Z.); 2 School of Public Administration, Central China Normal University, Wuhan 430079, China; zhangzuocug@163.com

**Keywords:** city-county merger, mediating effects, multi-period difference-in-differences method, urban energy intensity

## Abstract

The adjustment of administrative division is one of the powerful tools used to promote urbanization by the Chinese government in recent decades, and there is little literature to discuss whether the government-led expansion of urban space through city-county mergers can bring about a decline in urban energy intensity. With the multi-period difference-in-differences (DID) method and comprehensive urban datasets, this research investigates the urban energy intensity results of the city-county mergers policy experiment in China from 2000 to 2017. We present evidence that city-county mergers are indeed beneficial in reducing urban energy intensity, and that the energy-saving effect of the policy only starts to become significant in the third year after implementation. We also further adopt a series of robustness tests, such as the counterfactual test, placebo tests and PSM-DID tests to find if this effect still exists. The mechanism test with mediating effects indicates they are potential contributors to the positive effects of mergers with moderate fiscal centralization, population agglomeration and regional integration. We further explore positive effects of mergers, relying on the scientifically design official appraisal system and improve government efficiency.

## 1. Introduction

China’ s urbanization rate had exceeded 60% by the end of 2020, which indicates the process of economic development and the transfer of industrial structure to non-agricultural industries. It is also accompanied by the continuous increasing in the urbanization rate [1]. From the experience of developed countries, the core of urbanization is migration and agglomeration of labor and enterprises, while the expansion of urban space is the market-clearing equilibrium result of labor and land. The market mechanism is the main driving force for urban development in developed countries, and China’s rapid urbanization is characterized by government-led, large-scale and land-based public ownership [2]. Rapid urbanization has not only contributed to the expansion of cities but also brought about serious energy consumption. It is estimated that China’s urban economy will account for 90% of the country’s gross domestic product (GDP) by 2025, and the urban energy demand will account for 85% of the country’s total energy demand. Every 1% increase in the urbanization rate will require an additional consumption of 60 million tons of standard coal [3]. If energy consumption is not controlled, carbon emissions and air pollution (PM_2.5_, nitrogen oxides, sulfur dioxide and other air pollutants) will continue to worsen, and the annual economic cost of air pollution in China is estimated at 1.2% of GDP, which is based on the cost of disease, and it will rise to 3.8% if it is based on willingness to pay [4]. All of these factors mean that Chinese cities face the serious task of energy saving and consumption reduction, and it remains an open question whether government-led urban expansion can have a positive effect on reducing energy intensity.

**Figure 1:**
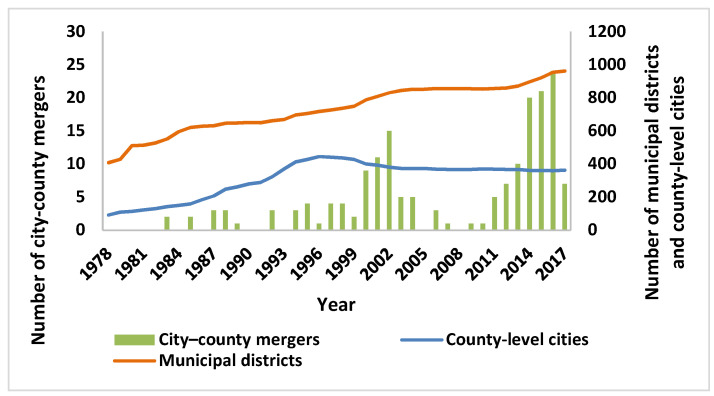
Number of municipal districts, county-level cities and city-county mergers since 1978.

China has implemented a series of administrative division adjustment policy after the reform and opening up in 1978, which aimed to promote industrialization and urbanization by adjusting interregional relations. In particular, the adjustment of administrative divisions has gradually become the main force in promoting the improvement of urbanization since the 1990s. Generally speaking, the increase in urbanization is mainly achieved through the growth of the number of cities and the expansion of the size of cities, both of which are closely related to the reorganization of administrative divisions in China: the creation of new cities (county-to-city upgrading) and the expansion of existing cities (city-county mergers). For the latter, city-county mergers are the abolition of counties (county-level cities) that were previously part of the prefecture-level city and the establishment of municipal districts in the administrative area of the former counties (county-level cities), thus achieve the purpose of expanding the size of the city. The small-town strategy was considered to be the most suitable urbanization path for China at the beginning of the reform and opening up in 1978 [5]; this policy did not achieve the expected goal of promoting urbanization and industrialization, although a large number of small cities and towns were newly established through county-to-city upgrading [6]. After the abolition of the county-to-city upgrading policy in 1997, the city-county merger policy began to be widely implemented. Are there also differences in the effects of these two types of policies in terms of reducing urban energy intensity? It has been a matter of debate which type of policy is more efficient between researchers and policy makers [7]. Unlike county-to-city upgrading, city-county mergers essentially expand the boundaries of existing cities and allow the population size of cities to rise further. A total of 119 prefecture-level cities and 174 counties (county-level cities) participated in city-county mergers during the period of 2000–2017. From Figure 1, we can see that there is a clear feature of uneven advancement of city-county mergers. Before 2000, the number of mergers was relatively small before 2000, and city-county mergers experienced two waves after 2000: the first wave was from 2000 to 2004, and there are 39 prefecture-level cities and 45 counties (county-level cities) which participated in the mergers. With the vigorous promotion of the new urbanization strategy, the second wave sprouted in 2011 and completely broke out in 2012–2017. A total of 78 cities have undergone 94 city-county mergers between 2011 and 2017. After this round of mergers, the number of Chinese cities has decreased, but the existing prefecture-level cities have realized a rapid expansion in population and land size, and the characteristics of population agglomeration in large cities have become more obvious. The city-county mergers will help optimize China’s urban space system and break down administrative boundaries between prefecture-level cities and the counties under their jurisdiction, which can achieve unified decision-making in urban energy planning.

City-county mergers have become an institutional choice for urban spatial expansion during a period of rapid urban development, with the aim of making effective use of economic scale and improving the scope and quality of public service provision [8]. City-county mergers essentially reflected in the state power based on a specific geographical space and the country’s political organization structure, which is used to carry out regional division or element allocation. The large-scale city-county mergers have also shaped space and governance characteristics in the long-term development of Chinese cities [9,10]. The reorganization of administrative districts to meet the needs of urban expansion is not unique to China; developed countries such as the UK, Germany and France have optimized urban management in the context of rapid urbanization by reorganizing their administrative districts since the 1960s. The success of city-county mergers in developed countries is subject to referendum by the local people and therefore reflects the needs of the people and the market. In contrast, the process of administrative redistricting in China is government-led that may run counter to the laws of the market and easily lead to pseudo-urbanization and urban sprawl, which will result in county resources being seized by prefecture-level cities. In a positive sense, merger policies may have promoted economic linkages and market integration between counties and city districts, which helps to reduce administrative barriers and enhance urban agglomeration economies, and also can accelerate population urbanization and economic development [11]. Therefore, whether the city-county mergers can promote the reduction in urban energy intensity remains to be tested empirically, which is the core motivation of this paper.

On the other hand, the state has gradually decomposed the binding targets for reducing energy intensity. starting with China’s 11th Five-Year Plan (FYP): the energy-saving indicators that reach the provinces are further decomposed into cities, and the cities further decompose the tasks into urban districts, so that urban districts become important units to implement the task of energy saving and consumption reduction. They must take a series of measures to achieve the energy intensity reduction targets, which are set in the Five-Year Plan. City-county mergers are almost irreversible policy and require the government to make scientific decisions and comprehensively evaluate various potential impacts on the urban energy intensity. The policy provides good target for examining whether government-led urban expansion through city-county mergers can reduce urban energy intensity, and the answers can help to assess the policy effects of the government-led green urbanization with city-county mergers. To our knowledge, there is a lack of in-depth research on this topic in the existing literature, and we attempt to fill this research gap by presenting this paper as the first empirical study about the impact of city-county mergers on urban energy intensity in the process of China’s rapid urbanization.

In this paper, we use city-level data from 2000–2017 to empirically analyze the urban energy intensity reduction effect of city-county mergers in Chinese cities. In line with most studies, we focus on city-county mergers after 2000 [7]. This is due to the fact that China’s urban development strategy has shifted from quantitative expansion to scale expansion with the abolition of county-to-city upgrading, and city-county mergers have been given a more important status and policy implications, which are more valuable to examine. The possible contributions of this paper include two main aspects. Firstly, the paper adopts the DID method to circumvent the possible endogenous problem in the empirical analysis, while at the same time systematically investigating the time-lagged effects of the impact of city-county mergers on urban energy intensity, which have practical significance for the proper understanding and appreciation of the policy effects of city-county mergers. Secondly, this paper focuses the impact of city-county mergers on urban energy intensity, while previous studies have mostly focused on economic growth and the efficiency of local public goods provision, and it identifies the mechanisms of merger effects on urban energy intensity from the perspective of local decentralization, agglomeration effect and regional integration. In general, we provide empirical evidence for evaluating the energy-saving performance of city-county mergers, which have led to a reduction in urban energy intensity to some extent. It comes from the formation of “active government” in reducing energy intensity, the energy-saving effect produced by spatial agglomeration, the free flow of factors and the optimal resources spatial allocation, thereby bringing about long-term performance of reducing energy intensity.

The remainder of the paper is organized as follows: Section 2 introduces the background and theoretical mechanisms of the urban administrative division system; Section 3 presents the econometric model and description of the relevant data; Section 4 gives the empirical results; Section 5 gives the impact mechanisms, and these are discussed in Section 6; Section 7 draws conclusions and policy implications.

## 2. Institutional Background and Literature Review

### 2.1. Institutional Background

#### 2.1.1. The Urban Districts Adjustment and Its Role in the Urban Development

**Figure 2:**
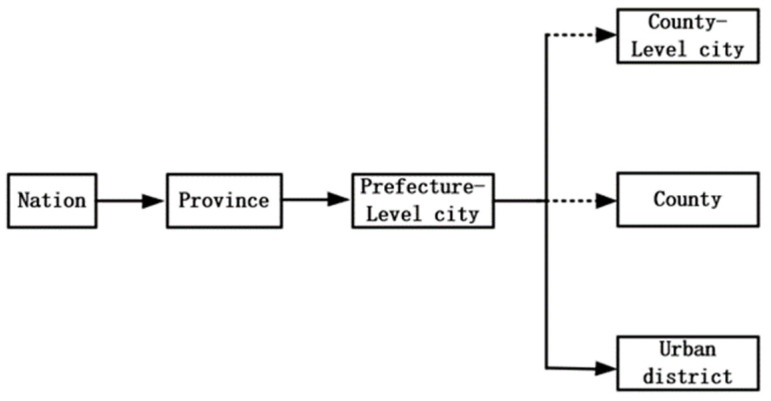
Administrative division of China. Source: Based on Tang and Hewings (2017) [8].

China currently consists of four main administrative hierarchies under a single system of government: national, provincial, prefectural-level city and county (see Figure 2). Chinese cities are characterized by distinct administrative hierarchy and cities with higher administrative levels having greater access to resources. This kind of urban administrative hierarchy established by administrative power is fundamental feature that distinguishes Chinese cities from those in Europe and the United States. Under this system, counties (county-level cities) and urban districts, whose are subordinated to prefecture-level cities have the same grade level and widely differing competencies between two levels of government. The main difference between them lies in their different discretionary powers over local affairs. The counties (county-level cities) under the jurisdiction of the cities are constitutionally subordinate administrative units with relative independence in terms of financial, administrative and human rights, while the municipal districts are under the direct jurisdiction of the prefecture-level cities and have relatively limited autonomy. The economic and transport links between urban districts have been greatly enhanced within the city jurisdiction, which leads to the urban districts becoming a unified body of urban economic and social policy [12]. Compared with urban districts, local governments in counties (county-level cities) have more autonomy in local affairs. In general, a significant change following city-county mergers is that coordination costs between municipal governments and the merged counties tend to be significantly lower. It remains to be tested whether the reduction in coordination costs brought about by the city-county mergers means that it is no longer sufficient to shake the reform dividends of the mergers and will not have a negative impact on the reduction in urban energy intensity. We follow the approach of Tang and Hewings (2017) and do not distinguish between counties and county-level cities in this study [8]. According to the Regulations on the Reform of China’s Administrative System, which is issued by China’s State Council in 1985, there are several steps in the implementation of city-county merger policy. The local governments (prefecture-level cities and counties) propose the mergers, which are reviewed and submitted by the provincial governments, the central government and the Ministry of Civil Affairs have the final approval or veto power.

The institutional basis of the restructuring of urban administrative districts is the city-county system and the enterprise model of local government formed by the decentralization reform. The fiscal decentralization reform devolved more development powers to local governments, and administrative divisions adjustments were given incentives and economic functions by the central to the local governments under the institutional framework of Chinese-style decentralization, profoundly changing the incentives and constraints of local officials, as well as the right scopes of local governments, the spatial structure of factors and even the competition pattern among local governments [13,14]. Usually, counties are rural in focus and their land use is much less efficient than urban districts, and the over-reliance on GDP as a relative performance assessment indicator has resulted in urban policies of economically inclined, municipal districts naturally becoming the focus of governance, which are urbanized economic zones under the direct jurisdiction of municipal governments. The rapid development of externally oriented economy and the tax-sharing system inspired by land finance and the corporatization of local governments have led to a strong demand for space in the more developed prefecture-level cities, and directly led to the expansion of municipal districts by city-county mergers as the most common method after 1997, which is similar to the “vertical integration” of corporate organizations, that is to say, a small-scale recentralization process under the framework of decentralization [15]. Although city-county mergers require the approval of the central government, local governments have the advantage of asymmetric information about their jurisdictions to put forward specific applications for the creation of districts, thus making the adjustment of administrative divisions also carry the economic and political demands of local governments. Driven by the “entrepreneurial spirit” of local governments, county governments are actively involved in the competition for regional resources and the protection of local markets. Even after the mergers, the interests of the city and county governments may be different at all levels, so the city-county mergers involve a game of interests between the prefecture-level cities and the counties (county-level cities) under their jurisdiction. In fact, the most important driving force of city-county mergers comes from the higher level of government, while the will of the counties (county-level cities) merged is less considered [16,17]. The above shows that officials have been excessively pursuing promotion indicators based on economic growth for a long time. With the assessment mechanism of local officials becomes more scientific by central government, the assessments of energy saving and consumption reduction as the core have made local governments politically motivated to be “accountable to superiors”, and the energy-saving performance of the city-county mergers has become the focus of stakeholders, especially the municipal governments. Whether the assessment, which mainly consists of the improvement of energy efficiency, really has a positive effect on the urban energy intensity still needs to be further tested.

#### 2.1.2. The Role of Urban District in Energy Saving and Consumption Reduction in the City

With resources and the environment becoming increasingly prominent bottlenecks to development, the Scientific Outlook on Development has been an important strategy as the political basis for environmental policy implementation since the 17th National Congress of the Communist Party of China, which places green and sustainable development in a prominent position. China began to address energy issues along with economic development since the 11th Five-Year Plan (FYP), which addresses specific goals including: (1) continuous improvement of physical energy efficiency and (2) transformation of the economic structure from high energy-consuming industries to low energy-consuming services. In line with these objectives, the central government has chosen economic energy intensity (energy consumption per unit of GDP) as the standard for setting targets. For example, it required that the energy consumption per unit of GDP should decline by 20% from the 2005 level in the 11th Five-Year Plan (FYP) for the first time. In the subsequent Five-Year Plan, the Chinese government set energy conservation targets as basic requirements for reducing energy consumption. In order to achieve the national energy intensity targets on schedule, on the one hand it depends on the scientific decomposition of the national targets into provinces, which are decomposed to cities in the provinces, and in turn further decomposes them to the urban districts. On the other hand, it depends on the effective implementation of decomposition indicators by local governments at all levels. 

The regional decomposition of energy intensity targets mainly follows the top-down approach; urban district governments develop relevant implementation plans, and the city government launches a strict target responsibility assessment to the district governments and strengthens the daily energy management to ensure the successful completion of the energy-saving goals. The specific responsibilities of district governments in terms of energy saving and consumption reduction include: strengthening energy-saving target responsibility, optimizing the energy structure, grasping the energy saving management of key energy-using units, implementing energy-saving key projects, energy conservation monitoring in accordance with the law and promoting green living concepts and lifestyles, etc. Due to the huge differences in the development stages and actual conditions, it is important to emphasize that the realization of energy-saving goals should take into account both the actual economic development and the potential of each district to achieve energy saving and consumption reduction. Therefore, the decomposition of the energy intensity target is mainly based on the principles of efficiency, fairness and feasibility, and the various allocation schemes are essentially different combinations of these principles, which make each urban district accomplish its own energy-saving target and at the same time lay an important foundation for the city to accomplish the overall target [18,19]. 

### 2.2. Literature Review

China’s administrative and economic zones exhibit a high degree of consistency and apparent conflict; it plays a very important role in resource allocation with both administrative and economic forces. City-county mergers may also have important impact on urban energy intensity by alleviating the inconsistency between the development of “administrative districts” and “economic districts”. It is generally accepted that city-county mergers may contribute to reducing urban energy intensity through the following three channels.

First, city-county mergers affect urban energy intensity by changing local government competition and the incentives of local officials under Chinese-style decentralization. A mixed incentive structure of economic decentralization and political democratic decentralization is core component of Chinese-style decentralization [20]. Liang et al. (2019) argue that city-county mergers are small-scale recentralization process within an overall decentralization framework, which reduces the number of local governments directly involved in competition. It also reduces the intensity of intergovernmental competition by reducing the autonomy and capacity of county governments, which will reduce productive expenditures and increase livelihood expenditures [21]. Bo and Cheng (2021) argue that transferring decision-making power of county government to prefecture-level municipalities gives greater priority to city governments, which form a more pronounced core-periphery structure at the prefecture-level cities; these results are driven by a reallocation of financial resources and industrial production based on productivity advantages and political favoritism [22]. Zhang L et al. (2018) believe that this is generally through a twofold mechanism of local government competition and local officials’ incentives and constraints with the impact of city-county mergers on the provision of public goods [23]. In terms of local government competition, Zhang C Y et al. (2018) find that it can lead to “healthy competition” in energy-saving governance with reasonable performance assessment indicators and a decentralization system. Specifically, energy efficiency performance indicators directly enhance the “race to the top” strategic interaction between local governments; economic performance indicators reduce the “race to the top” strategic interaction [24]. Song et al. (2018) believe fiscal decentralization can stimulate green total factor productivity (GTFP) growth, and this effect diminishes when the quantile increases, so that appropriate fiscal decentralization can improve GTFP while strong fiscal decentralization becomes a barrier to GTFP [25]. In terms of incentives and constraints for local officials, “green” criteria have been gradually incorporated into the performance assessment system of officials as the “Scientific Outlook on Development” has been comprehensively elaborated and promoted; the role of environmental regulations has changed from “constraints” to “facilitation” in improving regional energy efficiency [26,27]. The tenure of municipal party secretaries and mayors shows a significant inverted U-shaped relationship with expenditure on visible public goods such as rail transport and landscaping, and the highest point occurs in the third year. The significant relationship does not exist similarly for non-visible public goods, for example, gas and central heating [28]. 

Second, city-county mergers are accompanied by a concentration of population and factors that have an impact on urban energy intensity. Tang and Hewings (2017) found that city-county mergers can lead to significant population clustering and economic growth in prefecture-level cities [8]. Feng and Wang (2021) found that city-county mergers in eastern China could lead to 11.04% of urban expansion through resource integration, radiation and economic agglomeration, while it reached 16.17% and 10.20% in central and western China, respectively [29]. Morikawa (2012) argues that service sector establishments are more efficient in energy consumption in densely populated cities. After controlling differences between sectors, energy efficiency improved by about 12% when urban population density doubled. It would contribute to environmentally friendly economic growth in terms of relaxing excessive hinder urban agglomeration and investing in infrastructure in urban centers [30]. Otsuka et al. (2018) concluded that population density does have a positive impact on reducing energy intensity, and the impact varies by region. Population concentration in large urban districts reduces energy intensity, while population dispersion in rural areas increases energy intensity [31]. Yao et al. (2017) found the relationship between city size and electricity intensity appeared as an inverted U shape. With the growth in city size, urban electricity intensity has increased, and with city size expansion over the population threshold of 746.84 million urban electricity intensity decreases [32]. Lin et al. (2019) argue that when the degree of economic agglomeration is within a reasonable range, its growth can improve green economy efficiency because of the positive externalities. However, the emergence of negative externalities will harm green economy efficiency when the degree of economic agglomeration is too high [33]. 

Finally, city-county mergers have an impact on urban energy intensity through regional integration by reducing administrative barriers and promoting market integration. Under the Chinese decentralized system, the nature of the political promotion game embedded in economic competition leads to local protectionism and fragmentation of local markets with administrative districts as borders [34]. Gao (2011) argues that the reform of city-county mergers breaks down the rigid administrative barriers between urban districts and neighboring counties, which helps regional market integration and potentially improves resource allocation efficiency [35]. Qin et al. (2020) found that market segmentation in the thermal power sector fluctuated within a narrow range from 2006 to 2015 in most Chinese provinces and peaked between 2008 and 2010. The negative impact of market segmentation on environmental efficiency is both evident and strong, and the thermal power loss caused by environmental efficiency was as high as 15% in 2009 [36]. Li and Lin (2017) argue that regional integration has a significant and strong positive impact on energy and CO_2_ emission performance, with over 70% of the impact coming from man-made barriers rather than geographical distance [37]. Zhang et al. (2017) found that energy efficiency improved from 0.413 in 1986 to 0.739 in 2014 when market segmentation was taken into account, with an average annual growth rate of 2.1 percent. If the adverse effects of market segmentation could be removed, energy efficiency would gain an additional 1.5% per year on average [38]. It is worth noting that Luo et al. (2010) argue that the county government changes from independence to dependency of the city government after the city-county mergers, the pressure of assessment is reduced and the authority is correspondingly weakened, and the effect of incentive is significantly reduced. The spatial reorganization is difficult to leap into an organic market community, and may weaken the effective role of market in city-county mergers [16].

In summary, city-county mergers in China are a government-led process that is unlikely to fully reflect market demand, and it remains an open question whether it will ultimately reduce urban energy intensity or lead only to inefficient urban sprawl. The aim of this paper is to assess the net impact of city-county mergers on the reduction in urban energy intensity. It considers both the potential mechanism of merger effects on urban energy intensity and the trade-offs between the advantages and disadvantages. The strengthening of the city government’s ability to coordinate has effectively improved the efficiency of resource allocation between counties and districts after mergers [39]. It remains to be investigated whether strengthening ability to coordinate can smoothly integrate counties merged into the overall arrangement of urban energy saving and consumption reduction. There is little literature that has rigorously tested the effectiveness of this policy either theoretically or empirically. For this reason, we attempt to complete this work.

## 3. Data and Identification Strategy

### 3.1. Baseline Regression

The evolutionary characteristics of city-county mergers in China may provide a quasi-natural experimental advantage in examining its effects of energy-saving; we propose the DID approach to address this challenge. It is important to note that unlike the “one-size-fits-all” policy of uniform impact, the cities we studied do not have the same timing of city-county mergers. In contrast to the traditional DID model, we used the multi-period DID model which draws on Beck et al. (2010), and the basic model set-up as follows [40]:

(1) $$lnEI_{ct}=\alpha_{0}+\beta_{0}treated_{c}\times post_{ct}+\delta_{0}X_{ct}+\gamma_{t}+\nu_{c}+\varepsilon_{ct}$$

where *c* and *t* denote city and year, respectively, *lnEI_ct_* denotes urban energy intensity and is taken as logarithm and *treated_c_* is a dummy variable that responds to city-county mergers. If city *c* has occurred in 2000–2017, then take 1 (treatment group); if it has not occurred, then take 0 (control group). *Post_ct_* is a dummy variable for the year of policy implementation, and it takes the value of 1 in the year when the city implemented the mergers policy and after, otherwise it takes the value of 0. *X_ct_* is other control variable, $\gamma_{t}$ represents year fixed effects, $\nu_{c}$ represents city fixed effects and $\varepsilon_{ct}$ denotes the disturbance term. Obviously, *β*_0_ is the coefficient we focus on. If *β*_0_ is significantly less than 0, then it indicates that city-county mergers can reduce urban energy intensity, otherwise it indicates that it increases urban energy intensity.

### 3.2. Analysis of the Dynamic Effects of City-County Merger

To test whether the merger policy has long-term dynamic effects, we extend the model (1) to the following form:

(2) $$lnEI_{ct}=\alpha_{0}+\sum_{i=o}^{6}\beta_{i}treated_{c}\times post\left(i\right)+\delta_{0}X_{ct}+\gamma_{t}+\nu_{c}+\varepsilon_{ct}$$

where the variables are the same as in Equation (1), except that *post*(*i*) is redefined. *post*(*i*) is a dummy variable assigned to 1 in *i* year after merger and 0 for the rest (*i* = 0, 1, 2, …, 6). The main coefficients of interest are *β*_0_, *β*_1_, *β*_2_, …, *β*_6_. If *β*_0_ is significantly negative, it means that the urban energy intensity is effectively reduced in the year of implementation, and this reduction has gained instant results. If *β*_0_ is not significant and the coefficient of $treated_{c}\times post$ is significantly negative in a subsequent year, this indicates that the effect of merger on urban energy intensity has a significant lag.

### 3.3. Variable Description and Data Source

The dependent variable is urban energy intensity, which is obtained by dividing the urban energy consumption by the real urban GDP. Due to the lack of statistics on urban energy consumption, the conversion method is derived from Li et al. (2010); urban GDP is adjusted to the real GDP with 2000 as the base period [41]. Combining the main representative literature on the influencing factors of energy intensity, we selected the following variables as control variables. (1) Resource endowment (Endow). In the rich resource endowment area, the opportunity cost of energy access for local industries is relatively low, which makes it easy to cause resource allocation distortions in order to compromise energy efficiency. The share of urban extractive industry employees in the number of total employees is used to represent this variable [42]. (2) Industrial structure (Ind). China’s economic development still needs the pull of crude, energy-consuming industries in a longer period of time; industrial restructuring has a pull effect on energy intensity, which was measured with the ratio of gross industrial output value above designated size to GDP [43]. (3) Opening up (Open). The opening up has increased the international mobility of factors and reduced energy intensity through technology spillovers, which is represented with the ratio of real foreign investment to GDP in cities [44]. (4) Government dominance (Gov). Although the degree of influence of government dominance on the reduction in energy intensity has been gradually decreasing since the reform and opening up, there is still a partial loss of energy efficiency; we use the share of municipal fiscal expenditure in GDP to represent [45]. (5) Energy price (Price). The rise of energy prices can effectively reduce energy intensity through the effect of substitution between energy and other input factors, and we allocate the provincial fuel and power price index to each city in the province [46]. (6) Science and technology development (Tech). Science and technology development can make it possible to save energy input with the same output or to expand output with the same input, thus achieving an increase in energy efficiency. We use the share of the urban research and technical services employees in the total number of employees to express [47].

There were 298 Chinese prefecture and above prefecture-level (PAA) cities by the end of 2017 and there were only 263 cities in 2000. We selected 260 cities as our research objects, which have continuous and complete records over a period of 18 years. They account for 87% of the whole PAA cities in China and cover all the important cities, which include 4 municipalities directly under the central government, 15 sub-provincial cities and 30 provincial capitals, and there is no Lhasa city due to missing data. In the end, we used a panel dataset spanning 260 cities from 2000 to 2017. During sample period, 119 cities out of 260 cities underwent city-county mergers. Given the special status of municipalities directly under the central government, sub-provincial cities and provincial capitals, which are the focus of the central government’s regional development strategy and are given a special position to drive the development of the whole region [48,49]; above cities are excluded from sample in order to maintain the accuracy of the results. Based on the above treatment, we finally selected a sample of 225 prefecture-level cities (among which 97 cities underwent city-county mergers). Most of mergers took place in the year after the decree was issued, and some were only carried out in the following year after the decree was issued, and we record the actual year of occurrence. The main data sources of city-county mergers come from the “China Administrative Divisions Website” (http://www.xzqh.org/html/ (accessed on 8 July 2021)) and the Compendium of Administrative Divisions of the People’s Republic of China (2001–2018); part of the missing data was added through the provincial yearbooks. Fuel and power price indices were obtained from provincial statistical yearbooks (2001–2018), and primary data for the dependent and other control variables were obtained from the China Urban Statistical Yearbook (2001–2018). To avoid possible heteroskedasticity, the variables of resource endowment, government dominance and technological progress are treated as logarithms. Table 1 is the descriptive statistics of the main variables, which compare the differences between cities where the merger occurred and cities where the merger did not occur on these variables.

## 4. The Empirical Results

### 4.1. Baseline Regression Results

We used a multi-period DID approach to test the implementation effect of city-county merger policies; the results are presented in Table 2. Column (1) and (2) are the direct effects of city-county mergers on urban energy intensity, and column (1) represents the estimated results without the inclusion of control variables. It can be seen that the regression coefficient of the *treated_c_* × *post_ct_* is still negative and significant at the 1% level, providing preliminary evidence that mergers have significant contribution towards reducing urban energy intensity, and that the urban energy intensity is 17% lower than that of cities without city-county mergers. We can see that the impact direction of various variables on energy intensity is basically consistent with the findings of the current representative literature. In terms of the industrial structure, most Chinese cities are currently dominated by industry and still have a negative impact on reducing energy intensity. Opening up can have a positive impact on reducing energy intensity through industrial linkage and technology spillover effects. This caused higher energy intensity with a higher degree of local government intervention in economic activities, which precisely implies a loss of efficiency in resource allocation within the region. As new technologies, equipment and processes emerge, energy inputs can be saved for the same output. However, it is important to note that the technological progress is not entirely “green-biased”, and may lead to increased energy consumption as cities move in the direction of improving productivity and expanding production scale [50].

The baseline regression gives an estimate effect of city-county mergers, but it is not possible to give the evolution of the growth effect of mergers over time. To answer the above questions, we selected six periods in the year of the mergers and after, which not only helped to capture more comprehensively the marginal impact of city-county mergers, but also reflected the relationship between the urbanization process of mergers and the political promotion of local officials under the dual economic and political incentives. This is because officers generally serve a five-year term, and the six periods we chose can cover all the political cycles of officials at the time of the mergers. Column (1) and (2) in Table 3 examine the dynamic effects of city-county mergers, where column (2) are the estimated results considering the relevant control variables. Compared with non-merger cities, it can be found that the effect on urban energy intensity is not obvious in the year and the first two years after the implementation of merger policy, which indicates that there must be a time lag between the implementation of the merger policy and its effect. It may be due to the fact that it takes time to coordinate the tasks of energy conservation and consumption reduction and project preparation planning in the cities where the merger occurred. However, it is significantly negative at the 5% level from the third and subsequent three years after the policy implementation, indicating that the effect of the merger policy is gradual. The negligible effect in the first two years suggests that the positive impact of the policy on reducing energy intensity must be given sufficient time to take effect.

### 4.2. Robustness Test

Although the results with multi-period DID show that city-county mergers are indeed beneficial in reducing urban energy intensity, our DID strategy needs further verification. An important prerequisite for building a DID method is that the treatment and control groups have parallel trends prior to the implementation of merger policy, and it can lead to biased estimates under failure to meet this condition [51].

#### 4.2.1. Parallel Trend Test

**Figure 3:**
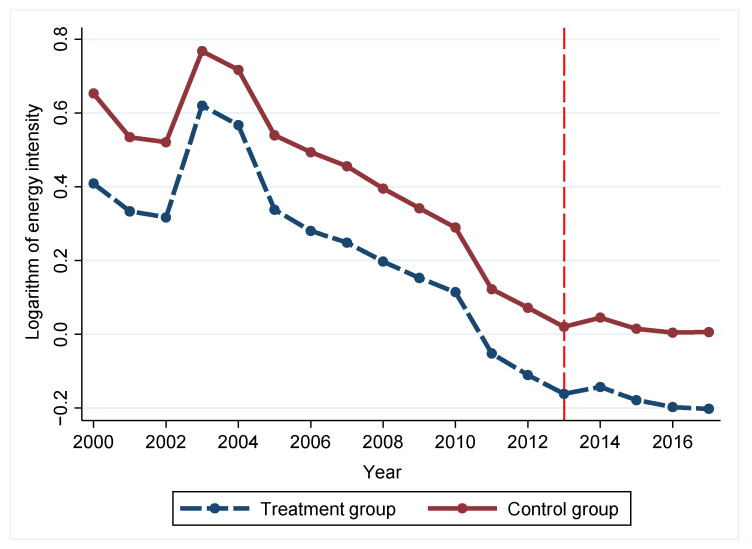
Parallel trend test.

We plotted the treatment group against the control group to illustrate the changes before and after mergers, which can be seen in Figure 3. It is difficult to depict the changes before and after mergers in different years in a single graph because the timing of mergers in the sample cities is not consistent. A total of 48 cities have undergone mergers up to 2013, which account for 49.5% of the treatment group. We refer to Wu et al. (2019) and use 2013 as the time of policy implementation for the analysis [52]. The energy intensity of the treatment and control groups basically showed parallel trends before 2013 and did not vary systematically over time, so the prerequisites for using DID were met.

#### 4.2.2. Full Sample Regression

Due to their special characteristics, municipalities directly under the central government, sub-provincial cities and provincial capitals were excluded in the basic regression. We used the 260 cities, which includes the above cities, to test the effect of mergers on urban energy intensity. Columns (1) and (2) show the regression results without and with control variables, respectively. It can be seen that the city-county mergers still significantly reduce urban energy intensity, and the coefficient is smaller than that in Table 2 (the coefficient of the explanatory variable is −0.170), indicating that cities with high administrative levels have a weaker effect on the energy intensity of their jurisdictions, as shown in Table 4.

#### 4.2.3. Counterfactual Test

Following Fan and Tian (2013), we conducted a counterfactual test by changing the timing of policy implementation [53]. In addition to the effect of city-county mergers on urban energy intensity, other policies or unobservable factors may also cause changes of urban energy intensity, and such differences may not be related to mergers and thus affecting the validity of the previous conclusions. In order to exclude the interference of such factors, we advanced the timing of mergers by 1, 2 and 3 years, and if the coefficient of effect of mergers was still significantly negative at this time, it indicated that the change of urban energy intensity was affected by other policy shocks or random factors, and not all of them were caused by mergers. Specifically, we advanced the policy shock by one year and set the variable pre(1) = 1, otherwise 0; two years in advance, pre(2) = 1, otherwise 0; and so on, introducing a total of three variables for 3 years in advance of the policy shock, and regressing the three variables instead of post_ct_. As can be seen in Table 5, the coefficients of *treated_c_ × pre (1)*, *treated_c_ × pre (2)* and *treated_c_ × pre (3)* were not significant after controlling for other variables, which indicates that the change of urban energy intensity is not caused by other factors but was due to city-county mergers.

#### 4.2.4. Placebo Test

**Figure 4:**
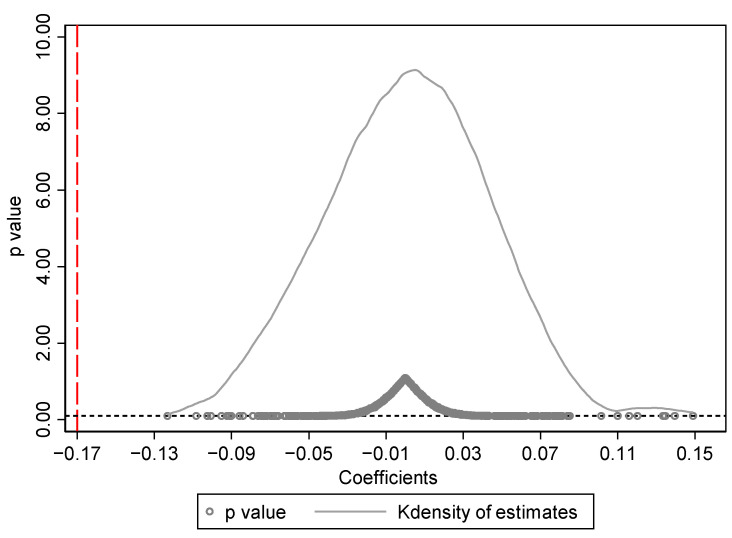
Placebo test.

We randomly selected 97 cities from 225 cities by the computer as the “pseudo-treatment group”, assuming that these 97 cities had implemented the merger policy and the other cities were the control group, and then generated “pseudo-policy dummy variables” for regression. Since the treatment and control groups are randomly assigned, we can expect the policy effect of the “pseudo-policy dummy variables” on urban energy intensity to be zero, otherwise the policy effect we obtained in the previous section is unreliable. At the same time, if merger policy really has a significant and positive effect on reducing energy intensity, we can expect the true estimated coefficient (−0.170) to be on the left of the placebo effect. It was repeated 500 times and the results are shown in Figure 4. We can see the estimated coefficients are mostly concentrated near the zero, and the p-values of most estimated values are greater than 0.1 (not significant at the 10% level), indicating that our estimate results are unlikely to be obtained by accident, and therefore are unlikely to be affected by other policies or random factors, which once again prove the robustness of the results.

#### 4.2.5. PSM-DID Test

DID can effectively identify the net effect of city-county merger policy and solve the endogenous problems but cannot overcome the problem of sample selection bias. Propensity Score Matching can solve this problem effectively under non-random experimental conditions. Therefore, we combined the DID method with this matching strategy, and the basic idea of the matching strategy was to find a similar control group of the treatment group. Specifically, we used the three-neighbor matching strategy and kernel matching strategy for the treatment and control groups on the basis of the control variables. We first constructed the matching balance test to test the reliability of matching results. The results of test show that there is no statistically significant difference in control variables between these two groups after the matching process. The estimation results of the PSM-DID are reported in Table 6. The results show that the effect of the merger policy on urban energy intensity is statistically significantly and negative, which once again proves that the merger policy does contribute to a reduction in urban energy intensity.

## 5. Analysis of Impact Mechanisms

Why city-county mergers show a more pronounced effect in reducing urban energy intensity is the interesting question considered in this section. As can be seen from the previous section, city-county mergers may affect urban energy intensity in three ways: the decentralization effect, agglomeration effect and regional integration effect. This section identifies and tests the above transmission pathways with the help of the mediating effects method. For fiscal decentralization (FD), the ratio of local general per capita budgeted fiscal expenditures to national general per capita budgeted fiscal expenditures is used for measurement [54]. For the agglomeration effect (POP), we used population density as its proxy variable, considering that rapid population agglomeration within urban space is a distinctive feature of urbanization [31]. As for regional integration (MS), we used market segmentation (total retail sales of consumer goods/GDP) as its reverse proxy variable [55]. The data on national general per capita budgetary expenditure were obtained from the China Financial Statistics Yearbook (2001–2018), respectively, and all other data were obtained from the China Urban Statistics Yearbook (2001–2018). We used the mediating effects to further analyze the possible paths of city-county mergers affecting urban energy intensity, which were proposed by Baron and Kenny (1986) [56]. The recursive model is constructed as follows:

(3) $$M_{ct}=\alpha_{1}+\beta_{1}treated_{c}\times post_{ct}+\delta_{1}X_{ct}+\gamma_{t}+\nu_{c}+\varepsilon_{ct}$$

(4) $$\ln EI_{ct}=\alpha_{2}+\beta_{2}treated_{c}\times post_{ct}+\mu_{2}M_{\mathrm{ct}}+\delta_{2}X_{\mathrm{ct}}+\gamma_{t}+\nu_{c}+\varepsilon_{ct}$$

where $M_{ct}$ represents the mediating effect, and the coefficient *β*_0_ of *treated_c_* × *post_ct_* in model (1) represents the total effect of city-county mergers on urban energy intensity. *β*_1_*μ*_2_ is the product of the coefficient *β*_1_ of *treated_c_* × *post_ct_* in model (3) and the coefficient *μ*_2_ of *M_ct_* in model (4), which represents the indirect effect of mergers on urban energy intensity, and the coefficient *β*_2_ of *treated_c_* × *post_ct_* in model (4) represents the direct effect of mergers on urban energy intensity. The total effect is equal to the sum of the indirect and direct effects, that is *β*_0_ = *β*_2_ + *β*_1_*μ*_2_.

Combining the sequential and bootstrap tests, the baseline regression model already reports the first step results of the recursive model (coefficient of the explanatory variable is −0.170), and Table 7 reports the second and third step results of the recursive model estimation, which confirmed the initial judgment of the second part of the mechanism analysis that mergers significantly reduces fiscal decentralization, market segmentation and increases population density, thus enabling mergers to reduce urban energy intensity through the channels of urban fiscal centralization, agglomeration effects and regional integration.

First, the fiscal centralization effect of city-county mergers is analyzed. The coefficient of the *treated_c_* × *post_ct_* in column (1) is significantly negative, suggesting that mergers can significantly reduce fiscal decentralization. Combined with column (4), we find that city-county mergers reduce urban energy intensity by reducing fiscal decentralization. This is consistent with the findings of Zhang et al. (2011) in that fiscal decentralization may be an important institutional source of declining regulatory standards and increasing energy consumption [57]; therefore, we argue that the focus of improving the current situation of high urban energy intensity is not to abandon the decentralization system but to strengthen its rationality. On the one hand, the fiscal centralization brought about by city-county mergers reduces local government competition, and on the other hand it may undermine incentives for local officials in counties merged, thereby reducing the efficiency gains from intergovernmental competition. Competition between district governments around the goal of reducing energy intensity in their own jurisdictions has to some extent contributed well to regional energy saving and consumption reduction, and excessive competition can also lead to ineffective allocation of resources and intensification of social conflicts. Improving the current performance appraisal system and introducing a multi-objective incentive mechanism will certainly make local governments more willing to pay attention to the effect of energy saving and consumption reduction while developing the economy, and establish a public service-oriented local government on the basis of maintaining the moderate enthusiasm of local governments, thus promoting the fundamental method for coordinating the tasks of energy saving and consumption reduction among urban districts. 

Second, we focus on the agglomeration effect of city-county mergers. The coefficient of the *treated_c_* × *post_ct_* in column (2) is significantly positive, indicating that mergers significantly increase urban population density, and the results in column (5) indicate that city-county mergers reduce urban energy intensity by increasing population density. It is not just a simple combination of regions, but city-county mergers increase the effective size of the city and improve the level of public services in the counties merged, and although it has a certain dilution effect on the public services of other urban districts, it promotes the agglomeration of elements throughout the city [58]. The externalities of energy saving and consumption reduction are considered as a “black box” of agglomeration, which can result in “self-cleaning” of negative environmental externalities [59]. Therefore, the positive externalities brought about by agglomeration, such as the reduction in transportation and information costs and the promotion of technological spillover effect among enterprises, can lead to the optimization of resource allocation and the improvement of utilization efficiency by directing the flow of resources from low-tech industries to high-tech industries within the region, thus promoting the development of high-tech industries and the elimination of low-tech industries, and reducing the energy intensity by correcting the distortion of enterprise factors and promoting the transformation and upgrading of the original industries. 

Third, we continue to explain the impact of the regional integration effect of city-county mergers on urban energy intensity. The estimation results in column (3) show that mergers significantly reduce the market segmentation of prefecture-level cities, indicating that mergers increase the level of regional integration, and the results in column (6) suggest that city-county mergers can reduce urban energy intensity by increasing regional integration. The municipal governments combine urban districts and the merged counties into one unit for urban planning, transportation system development, construction project approval and land supply, which provides an institutional basis for reducing administrative barriers and improving the transport spatial linkages between urban districts and the merged counties [60]. City-county mergers can not only improve market integration, but it can also eliminate the serious district government competitions and market fragmentation caused by the “administrative district economy”. Under the unified urban planning of municipal governments, expanding the radiation range of a city center while reducing district governmental frictions will improve the efficiency of resource allocation among the districts and county merged, including unified industrial layout, transport, communication networks and other infrastructure development, thus contributing to the reduction in the overall urban energy intensity.

## 6. Discussion

The current empirical literature on the policy effects of city-county mergers mainly focuses on the local public goods provision and economic development, and the results found that city-county mergers can increase the efficiency of local public goods provision and economic development [8,21,23]. It is rare within the literature to explore how such mergers reduce urban energy intensity in developing countries. This study further found that the city-county mergers have a positive impact on promoting urban energy intensity in China. This research not only provides a new understanding of the city-county mergers, but also provides valuable enlightenment for urban energy planning.

In addition, the DID method is one of the most popular methods in the field of policy evaluation, and is also used to evaluate the intertemporal effects of policy implementation [40,61]. Because it can better overcome the endogenous problem in the evaluation and its model settings are simple, many researchers are like to use the method to conduct policy evaluation. The previously mentioned scholars used the DID method to evaluate the merger policy on the supply of public goods and economic efficiency, such as Tang and Hewings (2017), Liang et al. (2019) and Zhang et al. (2018) [8,21,23]. The traditional DID method sets unified treatment group dummy variables and time dummy variables for the samples. The time for the city-county mergers to be studied in article is inconsistent in various places, which makes it unsatisfactory to meet the conditions of the traditional DID method. This requires the use of the multi-period DID method, which empirical strategy does not implement uniform treatment group dummy variables and time dummy variables; only the cross term of the two is included in the regression model and focuses on testing the sign and significance of the cross term.

Once the researchers need to empirically analyze the impact of a certain policy on changes in energy intensity, such as city development policies, they can use the DID method for analysis. We take the impact of city-county mergers on urban energy intensity as an example. First, we need to divide the sample into treatment group and control group. The treatment group is the city where merger occurred, and the control group is the city where the merger did not occur. Then, the regression method is used to examine the influence of the treatment group on the dependent variable, that is, the urban energy intensity. The results in this article reflect the effect of the merger policy, determined by the average gains of the treatment group after the policy change subtracted from average gains of the control group during the same period.

This research explores the influence of independent variables on dependent variables, obtaining the actual or theoretical relationship between them, and also attempts to further try to explore the internal mechanism or principle of the relationship; the mediating effect analysis provides the possibility to answer this question. Regarding the mechanism analysis of city-county merger effects, the use of mediation effects is the choice of many scholars in this field [39]. It can be seen from the analysis of the mechanism that the policy design for the city-county mergers does not completely directly affect the urban energy intensity. In addition to the direct merger effect on urban energy intensity, it also indirectly has a positive effect on reducing urban energy intensity through the fiscal centralization, agglomeration effects and the regional integration, and the total effect of the baseline regression result is the sum of the direct effect and indirect effect.

In the context of China’s decentralization system, the city-county mergers can bring about a small-scale recentralization process under the overall decentralization framework [15,21,22]. Consistent with the research by Zhang C Y et al., (2018), Song et al., (2018), Luo et al. (2017) and Zhang P et al. (2018) [24,25,26,27], we also found that moderate fiscal centralization, reduction in the competition intensity among governments and scientific incentives for local officials can have positive effects on reducing urban energy intensity. In addition, an important feature of the mergers is population agglomeration and expansion of the urban scale [8,29]. Consistent with the research by Morikawa (2012), Otsuka et al. (2018) and Lin et al., (2019) [30,31,33], we also found population agglomeration after merger can reduce urban energy intensity. In addition, city-county mergers can potentially increase the efficiency of resources allocation with strengthening the coordination and economic integration between governments [34,35,39]. The regional integration brought about by the mergers is conducive to the improvement of urban energy intensity, which is consistent with related research results by Qin et al., (2020), Li and Lin, (2017) and Zhang et al. (2017) [36,37,38].

Compared with the optimal size, it should be noted that previous studies believe that the size of Chinese cities is generally small [62]. However, Yao et al. (2017) believe that the relationship between city size and electricity intensity appeared as an inverted U shape [32], meaning that our results indicate that many Chinese cities may reach or exceed the optimal scale. The results do not mean that urban energy intensity can be improved only through the adjustment of administrative divisions such as city-county mergers. Although we have confirmed the positive effects of mergers that occurred in 2000–2017, it depends on two conditions: scientific design and improvement of a diversified official appraisal system, and effective reduction in jurisdictional barriers and improvement of government efficiency. Facing both economic and political incentives for local governments, we found that the merger effects have a significant positive effect on reducing urban energy intensity from the third and subsequent three years after the policy implementation. On the one hand, it shows that the assessment which mainly consists of the improvement of energy efficiency already has some positive effect in reducing urban energy intensity. On the other hand, there is not an inverse U-shaped relationship between city officials’ tenure and non-visible expenditures, such as scale of gas and central heating (Wu et al., 2018), which indicates that a “non-visibility bias” will lead to a strategic response from officials [28]. The central government should redesign performance evaluation systems to rebalance the incentives of local officials; it is important to establish an accountability mechanism that spans the tenure of officials, and officials should be held accountable even after they step down. In addition, the spatial reorganization is difficult to leap into an organic market community and may weaken the effective role of market in city-county mergers [16]; whether the coordination costs can be reduced and government efficiency can be truly improved depends on the intensity and effect of the merger reform. Under these backgrounds, it is still remains to be seen whether the implemented policy of city-county mergers can achieve the expected goals and effectively promote the decline in urban energy intensity.

## 7. Conclusions and Policy Implications

Based on the above analysis, how city-county mergers reduce urban energy intensity in China has rarely been studied. We examined the impact of city-county merger policy on urban energy intensity in China, which not only provides new insights about the effect of mergers, but also provides valuable insights about urban energy planning. China is experiencing the largest rural-to-urban migration in the history of the world, and the Chinese government has set a clear binding target of reducing energy consumption per unit of GDP from the 11th to 13th Five-Year Plan. With the promotion of new-type urbanization and energy saving and consumption reduction becoming the two main themes in the green transformation of China’s economy, it is an urgent problem facing Chinese policymakers with establishment of a green development urban space system. In this context, the Chinese experience of promoting merger policy can be used as reference for many developing countries facing rapid urbanization.

City-county mergers are one of the key tools of China’s urbanization strategy, and such policy experiments provide a good research object for testing whether government-led urban expansion promotes the decline in urban energy intensity. While urban district governments are required to carry out special inspections and report to the municipal government on energy saving and consumption reduction every year due to the decomposition of energy-saving constraint indicators, it has rarely been discussed whether the situation of city-county mergers has an impact on urban energy intensity, which is an important theoretical and practical issue in China’s new-type urbanization strategy. We find that city-county mergers have a positive effect on the reduction in urban energy intensity, which starts to have a significant effect in the third year after implementation. The results have passed a series of robustness tests and provided a reasonable explanation from regional decentralization, agglomeration effect and regional integration, which are combined with the intermediary effect. City-county mergers are a spatial reorganization of the transformation of county economies into urban economies; the task of energy saving and consumption reduction can only be accomplished through the comprehensive and unified coordination of the prefecture-level city government from top to bottom. The positive effect of the mergers also relies on the scientific design and improvement of the diversified official appraisal system, effectively reducing jurisdictional barriers and improving government efficiency.

The above findings bring a lot of important enlightenments. They provide an important reference for other prefecture-level cities that are planning to achieve “win-win” of economic growth and energy saving through city-county mergers. It is worth noting that although the city-county mergers can help achieve energy saving and consumption reduction, it will inevitably take time to achieve the intended goals.

Although city-county mergers are conducive to the coordination of energy saving and consumption reduction by city governments, there is still a need to provide effective incentives to local officials of counties merged. The reform of city-county mergers is not “free lunch”, and it must face the large coordination costs between cities and counties merged. Municipal governments must decentralize power appropriately to ensure the weight of energy-saving component of the performance evaluation indicators while centralizing fiscal power; it is important to hold the counties merged government to participate in the energy-saving and consumption-reduction tasks as the top priority. The setting of performance assessment indicators should not follow the old path of sacrificing energy consumption for rapid economic growth, and it also cannot only pursue energy conservation at any economic cost. As a “hard constraint”, energy-saving performance indicators should be accompanied by more financial and administrative powers to encourage the “healthy competition” of energy-saving governance among urban district governments, so that the implementation of merger policy can achieve the twice the result with half the effort effect. In addition, the assessment of energy conservation and consumption reduction should highlight normalization and differentiated assessments, which can establish the leading goose effect in similar assessments and form active government in reducing energy intensity.

In order to achieve the energy saving and consumption reduction targets as early as possible, we need to appropriately relax administrative intervention policies that excessively restrict economic and population clustering. In the process of city-county mergers, municipal governments should encourage to introduce effective policies to actively promote the rational clustering of various economic activities within cities, and promote the urban development in the direction of specialization, industrialization and agglomeration. Taking into account the balance of polarization and diffusion effects, city-county mergers should achieve positive mutual promotion with urbanization; on the one hand, energy intensity can be reduced through industrial structure upgrading with intensive urbanization layout, strengthening infrastructure construction and green knowledge spillover. On the other hand, the agglomeration effects can reduce the cost of information transmission, improve enterprise production efficiency and promote the formation of economies of scale, making it play an important role in reducing energy intensity. While expanding the scale of the cities through city-county mergers, attention should be paid not only to preventing excessive agglomeration, but also to reduce the negative effects caused by the inefficiency of “urban sprawl”. The control of the urban size and density should take into account the differences in the environmental carrying capacity of different cities, avoiding setting uniform standards based on the existing classification of Chinese cities’ tiers, and impose a limit on the cities with existing population sizes exceeding the optimal sizes based on energy efficiency. For cities whose population size is below the optimal population size, they should give full play to their population absorption potential.

Finally, regional integration can improve energy saving and consumption reduction by promoting the free flow of factors and optimizing the efficiency of resource space allocation, which is an important tool to promote coordinated regional economic development. However, energy-intensive enterprises within cities tend to migrate to urban boundaries and areas with low environmental barriers to entry [63]. In order to avoid the threat posed by the transfer of high energy consumption in the integration process to the completion of energy-saving and consumption reduction tasks in counties merged as much as possible, the formulation of criteria for city-county mergers that match the stage of economic development and strict merger procedure will not only help the city-county mergers to break down administrative barriers to drive regional market integration, but also help strengthen the cooperation of municipal district governments in energy saving and consumption reduction during the integration process. By further strengthening the institutional arrangements for integrated regional development strategies between municipal districts, it will promote a synergistic mechanism for energy saving and consumption reduction policies, which can further break market segmentation and create an “effective market”, that is to say, accelerating the flow and diffusion of economic factors among neighboring districts and promoting a mutual reduction in energy intensity. By striving to form an effective interregional connection in related energy policies such as enterprise energy-saving subsidies, new energy industry support and incentives for the application of new energy technologies, this not only can provide basic guarantees for the realization of overall energy-saving goals, but also could be an important way to achieve the goal of reducing energy intensity.

The shortcomings of this paper lie in several points. It is still necessary to compare and analyze the economic strength of merged counties. Strong counties can still maintain their relative independence after merger policy is implemented, and this will have adverse effect for coordinating the task of energy conservation and consumption reduction. In addition, there are also differences between the active adaption and passive adjustment of city-county mergers; the former is actively implemented based on the needs of urban development and overall planning, while the latter is accompanied by undoing prefecture and setting up prefecture-level city, and what impact they will have on energy intensity is a topic worthy of further study. There are counties merged that are further away from the city center, which tends to make the support and supervision of energy conservation governance from the municipal government relatively small. Therefore, it will help to examine the local government’s energy conservation and consumption reduction strategies with analysis of the formation and mechanism of the boundary effect on energy conservation and consumption reduction, which is based on the spatial and geographical characteristics of cities under the pressure of energy conservation assessment. We noted that the effect of mergers occurs in the third year, so the energy-saving effects of regional decentralization, agglomeration effects and regional integration are all delayed, and it will also be an important part of our future work with systematic quantification of the impact mechanisms as well as heterogeneity. Due to the above reasons, the impact of city-county mergers on urban energy intensity is still complex and the effect of mergers should be evaluated carefully.

## Figures and Tables

**Table 1:** Descriptive statistics of the main variables.

Variables	Treatment Groups (97) (Cities Where Merger Occurred)		Control Groups (128) (Cities Where Not Merger Occurred)	
	Mean	Standard Deviation	Mean	Standard Deviation
lnEI	0.162	0.505	0.395	0.609
Ida	1.540	1.007	1.377	0.792
lnEndow	−4.557	2.078	−3.905	2.107
lnGov	−1.987	0.670	−1.796	0.607
Price	104.784	8.633	104.945	8.841
Open	0.037	0.060	0.029	0.038
lnTech	−4.181	0.835	−4.304	0.841

**Table 2:** Baseline regressions.

Variables	(1)	(2)
*treated_c_* × *post_ct_*	−0.250 ***	−0.170 ***
	(−19.23)	(−12.14)
lnEndow		0.050 ***
		(25.01)
Ind		0.033 ***
		(5.27)
Open		−2.027 ***
		(−13.88)
lnGov		0.039 ***
		(3.94)
Price		0.001
		(1.22)
lnTech		−0.067 ***
		(−11.17)
Constant	−0.018 ***	−0.115
	(−2.95)	(−0.93)
City fixed effect	Yes	Yes
Year fixed effect	Yes	Yes
Observations	4050	2981
R^2^	0.511	0.549

Note: *** represent the significance levels of 1%. t statistics in parentheses.

**Table 3:** Regression results of dynamic effects on energy intensity.

Variables	(1)	(2)
*treated_c_* × *post* (0)	−0.061	−0.067
	(−1.36)	(−1.40)
*treated_c_* × *post* (1)	−0.065	−0.063
	(−1.38)	(−1.57)
*treated_c_* × *post* (2)	−0.061	−0.062
	(−1.42)	(−1.22)
*treated_c_* × *post* (3)	−0.194 ***	−0.183 ***
	(−4.13)	(−3.98)
*treated_c_* × *post* (4)	−0.204 ***	−0.125 **
	(−3.64)	(−2.12)
*treated_c_* × *post* (5)	−0.208 ***	−0.137 ***
	(−3.25)	(−3.21)
*treated_c_* × *post* (6)	−0.205 ***	−0.147 **
	(−3.06)	(−2.19)
Other control variables	No	Yes
Constant	−0.060 ***	−0.080
	(−3.37)	(−0.67)
City fixed effect	Yes	Yes
Year fixed effect	Yes	Yes
Observations	4050	2981
R^2^	0.511	0.549

Note: ** and *** represent the significance levels of 5% and 1%, respectively. t statistics in parentheses.

**Table 4:** Regression with full sample.

	(1)	(2)
*treated_c_* × *post_ct_*	−0.287 ***	−0.165 ***
	(−28.17)	(−15.11)
Other control variables	No	Yes
Constant	0.585 ***	0.659 ***
	(34.41)	(6.16)
City fixed effect	Yes	Yes
Year fixed effect	Yes	Yes
Observations	4680	3501
R^2^	0.525	0.556

Note: *** represent the significance levels of 1%. t statistics in parentheses.

**Table 5:** Estimation results of counterfactual test.

	(1)	(2)	(3)
*treated_c_* × *pre* (1)	0.033		
	(0.79)		
*treated_c_* × *pre* (2)		0.012	
		(0.26)	
*treated_c_* × *pre* (3)			0.021
			(0.43)
Other control variables	Yes	Yes	Yes
Constant	0.579 ***	0.578 ***	0.575 ***
	(4.08)	(4.19)	(4.29)
City fixed effect	Yes	Yes	Yes
Year fixed effect	Yes	Yes	Yes
Observations	2981	2981	2981
R^2^	0.521	0.552	0.524

Note: *** represent the significance levels of 1%. t statistics in parentheses.

**Table 6:** Estimation results of PSM-DID.

	(1)	(2)
*treated_c_* × *post_ct_*	Neighbor matching	Kernel matching
	−0.139 ***	−0.169 ***
	(−9.27)	(−12.07)
Other control variables	Yes	Yes
Constant	−0.120	−0.122
	(−0.78)	(−1.05)
City fixed effect	Yes	Yes
Year fixed effect	Yes	Yes
Observations	1412	2977
R^2^	0.592	0.546

Note: *** represent the significance levels of 1%. t statistics in parentheses.

**Table 7:** Analysis of impact mechanisms.

	(1)	(2)	(3)	(4)	(5)	(6)
	lnFD_ct_	lnPOP_ct_	MS_ct_	lnEI_ct_	lnEI_ct_	lnEI_ct_
*treated_c_* × *post_ct_*	−0.047 ***	0.012 ***	−0.073 ***	−0.168 ***	−0.167 ***	−0.180 ***
	(−2.94)	(2.81)	(−5.21)	(−12.14)	(−11.93)	(−12.15)
lnFD_ct_				−0.040 ***		
				(−4.10)		
lnPOP_ct_					0.011 **	
					(2.25)	
MS_ct_						−0.138 ***
						(−17.28)
other control variables	Yes	Yes	Yes	Yes	Yes	Yes
Constant	0.505 ***	6.362 ***	2.284 ***	0.503 ***	0.415 ***	0.899 ***
	(3.14)	(35.71)	(15.33)	(4.16)	(3.14)	(7.13)
City fixed effect	Yes	Yes	Yes	Yes	Yes	Yes
Year fixed effect	Yes	Yes	Yes	Yes	Yes	Yes
Observations	2975	2952	2980	2975	2952	2980
R^2^	0.570	0.231	0.345	0.548	0.556	0.550

Note: ** and *** represent the significance levels of 5% and 1%, respectively. t statistics in parentheses.

## Data Availability

Publicly available datasets were analyzed in this study. The yearbooks data can be found here: [http://www.stats.gov.cn/, accessed on 10 July 2021].

## Abbreviations

DID	Difference-in-Differences
PSM-DID	Propensity Score Matching Difference-in-Difference
GDP	Gross Domestic Product
PM_2.5_	Particulate Matter less than 2.5 Micrometers in Diameter
FYP	Five-Year-Plan
GTFP	Green Total Factor Productivity
CO_2_	Carbon Dioxide
PAA	Prefecture and Above prefecture-level
